# Identification of Candidate Tolerogenic CD8^+^ T Cell Epitopes for Therapy of Type 1 Diabetes in the NOD Mouse Model

**DOI:** 10.1155/2016/9083103

**Published:** 2016-03-16

**Authors:** Cailin Yu, Jeremy C. Burns, William H. Robinson, Paul J. Utz, Peggy P. Ho, Lawrence Steinman, Alan B. Frey

**Affiliations:** ^1^Department of Cell Biology, New York University Langone School of Medicine, 550 First Avenue, New York, NY 10016, USA; ^2^Division of Immunology and Rheumatology, Department of Medicine, Stanford University School of Medicine, Stanford, CA 94305, USA; ^3^Geriatric Research Education and Clinical Center, Veterans Affairs, Palo Alto Health Care System, Palo Alto, CA 94304, USA; ^4^Department of Neurology and Neurological Sciences, Stanford University School of Medicine, Stanford, CA 94305, USA

## Abstract

Type 1 diabetes is an autoimmune disease in which insulin-producing pancreatic islet *β* cells are the target of self-reactive B and T cells. T cells reactive with epitopes derived from insulin and/or IGRP are critical for the initiation and maintenance of disease, but T cells reactive with other islet antigens likely have an essential role in disease progression. We sought to identify candidate CD8^+^ T cell epitopes that are pathogenic in type 1 diabetes. Proteins that elicit autoantibodies in human type 1 diabetes were analyzed by predictive algorithms for candidate epitopes. Using several different tolerizing regimes using synthetic peptides, two new predicted tolerogenic CD8^+^ T cell epitopes were identified in the murine homolog of the major human islet autoantigen zinc transporter ZnT8 (aa 158–166 and 282–290) and one in a non-*β* cell protein, dopamine *β*-hydroxylase (aa 233–241). Tolerizing vaccination of NOD mice with a cDNA plasmid expressing full-length proinsulin prevented diabetes, whereas plasmids encoding ZnT8 and D*β*H did not. However, tolerizing vaccination of NOD mice with the proinsulin plasmid in combination with plasmids expressing ZnT8 and D*β*H decreased insulitis and enhanced prevention of disease compared to vaccination with the plasmid encoding proinsulin alone.

## 1. Introduction

Several dozen autoantigens related to type 1 diabetes have been described [1] engendering interest in developing an immunotherapeutic treatment. Phase III clinical trials in newly diagnosed patients using noncomplement fixing anti-CD3 Ab failed to achieve primary endpoints [2]. Together with heightened risks to patients receiving any type of systemic immunosuppression, this setback for nonspecific immunological control of disease progression in patients emphasizes the impetus to explore optimization of antigen-specific immunotherapy for prevention and treatment of type 1 diabetes [3–5]. Experimental immunotherapies that target individual antigens have been tested [6] but which have also proven disappointing; phase III GAD vaccination trials failed [7, 8] and preclinical studies targeting Hsp60 (p277) showed some disease protection [9] but a late stage clinical trial study was retracted [10]. More promisingly, preclinical [11] and phase I/II [12] trials using tolerogenic DNA plasmids encoding proinsulin showed encouraging improvement in subject C-peptide levels.

Since CD8^+^ T cells play a causal role in diabetogenesis [13] an important goal in development of an effective immunotherapy is identification of specific epitopes that elicit diabetogenic CD8^+^ T cells. A substantial number of candidate MHC Class I epitopes have been identified in both the NOD mouse [14] and patients [1] including multiple peptides derived from *β* cell antigens that elicit robust Ab response in patients as well as other candidate antigens not exclusively expressed in *β* cells [15, 16]. Humoral responses are successfully used clinically to forecast disease susceptibility [13, 17], and since adaptive B cell response usually requires T cell help, the presence of reactive Ab in patients implies the involvement of CD4^+^ T cells.

Study of the NOD model has revealed a temporal pattern in development of T cell responses: circulating or islet-infiltrating CD8^+^ T cells reactive to certain antigens are detected at different ages and disease status [3]. Attempts to induce antigen-specific tolerance in diabetogenic CD8^+^ T cells aim to develop therapies to treat patients such that *β* cell function recovers, which might be replaced via endogenous stem cells or exogenous allograft transplants. In spite of abundant representation in young prediabetic mice, T cells specific for glutamic acid decarboxylase 65 (GAD65) or islet-specific glucose-6-phosphate catalytic subunit-related protein (IGRP) appear to not be dominant in type 1 diabetes initiation since mice tolerant to those antigens develop disease [18, 19]. However, induction of tolerance to insulin [20] or proinsulin [11] in young mice prevents development of both T cells reactive with other *β* cell antigens and disease progression therein implicating immune response to insulin as the major initiating event in type 1 diabetes [20]. Since noninsulin antigens likely contribute to development or expansion of clinical disease and thus represent candidate therapeutic targets for tolerance induction, we have sought and identified CD8 epitopes that may contribute to optimal tolerization in type 1 diabetes.

## 2. Research Design and Methods

*Mice*. NOD/ShiLtj female mice (The Jackson Laboratory, Bar Harbor, ME) were housed five per cage in a pathogen-free barrier facility and were maintained on a 12 hr light/dark cycle (7 AM to 7 PM) with* ad libitum* access to autoclaved food and water. Experiments involving animals were conducted with the approval of the New York University School of Medicine IACUC (protocol # 150219). Penetrance of diabetes in females is 90% at 32 weeks of age.

*Peptides*. Purity of peptides (Lifetein, South Plainfield, NJ) used in proliferation and IFN-*γ* assays was >85% and that used in tolerance assays was 99%. Peptides were solubilized in sterile PBS and 0.1 mg/mL of each peptide was analyzed for LPS (Lonza, Allendale, NJ). Any peptide having >0.1 EU/mg LPS was treated to remove endotoxin (Thermo Scientific, Waltham, MA) until samples were <0.1 EU/mg (1 EU = 0.1 ng endotoxin).

*Epitope Prediction Analyses*. For the SYFPEITHI analysis (http://www.syfpeithi.de/) the naturally presented epitope is expected to be within the top 2% of peptides predicted assuming 80% reliability. RANKPEP analyses (http://imed.med.ucm.es/Tools/rankpep.html) were performed with the “immunodominance filter” being off and “proteasome cleavage filter” being on. For the RANKPEP analysis binding thresholds for the candidate epitopes are as follows: IGRP > 17.9% for D^b^ and > 19.2% for K^d^, D*β*H > 17.9 for D^b^ and > 14.5% for K^d^, insulin > 17.9% for D^b^ and > 14.5%  K^d^, Gad65 > 17.9% for D^b^ and > 14.5 for K^d^, ICAp69 > 17.9% for D^b^ and > 14.5% for K^d^, ZnT8 > 17.9% for D^b^ and > 14.5% for K^d^. The IEDB analysis (http://www.iedb.org/) was performed using settings: “default prediction method,” the “immunoproteasome cleavage prediction type,” and the “TAP transport prediction.”

A known MHC Class I K^d^ binding peptide was synthesized and used as control [Influenza NP_147–155_ (TYQRTRALV) [21]]. There were a total of 69 peptides synthesized including previously characterized epitopes in insulin_(39–47 V)_ [22], IGRP_(206–214)_, IGRP_(251–259)_ [23], GAD65_(178–186)_, and GAD65_(546–554)_ [24] intended to serve as positive controls. The insulin epitope was prepared with a G>V substitution in the last position since G binds poorly to the pocket F [22].

*Antibodies and Flow Cytometry*. Antibodies (anti-K^d^ (SF1-1.1.1), anti-D^b^ (28-14-8), and anti-IFN*γ* (R4-6A2)) were purchased from eBioscience (San Diego, CA) and used as described previously [25, 26]. Analyses were performed on a FACS Calibur flow cytometer (Becton-Dickinson, Palo Alto, CA).

*IFNγ Assay*. Supernatants of 2 × 10^5^ cells cultured for 48 h with 10 *μ*M peptide (from 2-3 pooled mice PLN) were assayed by ELISA using 3,3′,5,5′-tetramethylbenzidine as substrate (eBioscience, # 88-8314-88, minimum sensitivity = 0.7 pg/mL). Data is shown as mean ± SEM.

*Proliferation Assay*. Single-cell suspensions were prepared and analyzed in quadruplicate wells using 96-well plates by measurement of [^3^H]-thymidine incorporation after stimulation with peptides as indicated or as positive control plate-bound anti-TCR*β* (H57-597) using 2 × 10^5^ LN cells/well [27]. Assays contained 5 × 10^5^/well mitomycin C-treated splenocytes as APC. Wells receiving Flu NP_147–155_ peptide served as negative control. Stimulation with anti-TCR*β* gave incorporation typically >10–20 times greater than nonpulsed wells. Incorporation of thymidine into nonpulsed wells varied in experiments and ranged from ~200 to ~900 cpm. Incorporation of thymidine into wells pulsed with control Flu NP_147–155_ peptide varied in experiments and ranged from ~400 to ~1,100 cpm. Thus, the average thymidine incorporation at a given peptide concentration was divided by average incorporation into wells pulsed with Flu NP_147–155_ and shown as “fold stimulation” in order to take into account this variation. Error bars represent standard error of the four wells at each peptide concentration tested for a representative assay.

*MHC Class I Stabilization Assay*. RMA-S cells expressing both D^b^ and K^d^ [28–30] (from M. Bevan, Univ. Washington, Seattle, WA) were maintained in complete RPMI-1640 medium containing G418 (Biowhittaker, Walkersville, MD). Cells were cultured at 26° overnight, pulsed with candidate peptide (10^5^–10^10^ M) for 60 min at 26°, incubated at 37° for 4 h, and assayed by flow cytometry for cell surface expression of D^b^ and K^d^. Assays were performed more than three times for each peptide.

*Tetramer Analysis*. Fluorochrome-conjugated tetramers were prepared by the NIH Tetramer Core Facility at Emory University (Atlanta, GA). Cells were stained with APC-conjugated anti-CD8 simultaneously with Alexa 488-conjugated tetramer, or PE-conjugated CD8 and APC-conjugated tetramer (1 : 100–1 : 200 dilution). Flow cytometry data was analyzed using FlowJo (Tree Star, Ashland, OR). Assays were performed more than three times for each peptide.

*Plasmid Construction*. The plasmid pBHT-568 expresses the entire murine proinsulin (lacking the signal sequence) and thus contains the dominant epitope residues 39–47 [11]. The proinsulin sequence was removed and full-length cDNAs encoding ZnT8 and encoding separately D*β*H (both from Open Biosystems) were cloned into. Plasmids were purified (EndoFree Giga Kit # 12391, Qiagen Corp, Valencia, CA) followed by removal of residual endotoxin as for synthetic peptides.

*High Zone Tolerance*. Peptides were injected i.p. in 0.2 mL final volume. Mice were injected at weeks 4, 6, 8, 11, and 14. This experiment was performed once (*n* = 40 mice per group).

*Zymosan A Tolerance*. Zymosan A (Sigma Aldrich, St. Louis, MO, # Z4250) was prepared as described [31]. 2 mg was mixed with 0.1 mg peptide in a final volume of 0.2 mL sterile PBS and injected i.p. The control group received Zymosan plus NP_147–155_. This experiment was conducted twice using 10 mice per group and the data were combined for statistical analysis.

*DNA Plasmid Tolerance*. Four-week-old mice were injected weekly with 0.025 mg of individual plasmids dissolved in PBS + 0.9 mM CaCl_2_ into each quadriceps in 0.1 mL (0.05 mg plasmid/injection/mouse) for a total of 6 injections. This experiment was conducted twice using 10 mice per group and the data were combined for statistical analysis. Control mice received injection of the empty vector.

*Histology*. Pancreata were fixed overnight in 10% buffered formalin before embedding in paraffin and processed for H/E staining. 10-micron sections were made of the whole organ and slides were scored in a blinded manner for both the number of islets per pancreas and the extent of islet infiltration (0%, 1–25%, 26–50%, 51–75%, and >75%). At least 100 islets per treatment group were analyzed.

*Glucose Monitoring*. Tail vein blood glucose was tested using OneTouch Ultra Test Strips and Monitor (Lifescan, Shelton, CT). Values greater than 250 mg/dL were considered positive. The log-rank test (Mantel-Cox) was used for statistical analysis of diabetes incidence at 30 weeks (GraphPad Software, San Diego, CA).

## 3. Results

**Figure 1:**
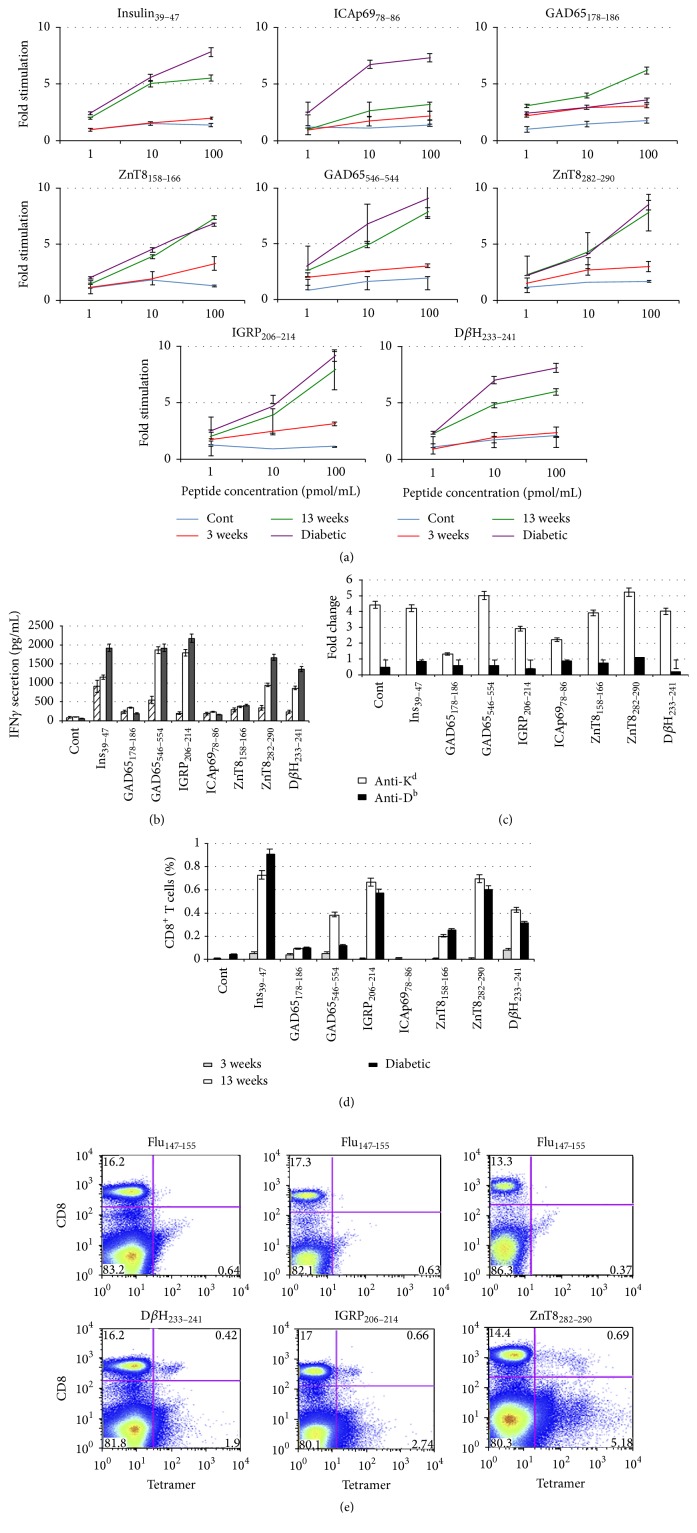
(a) Proliferation assay of PLN cells. 2 × 10^5^ LN cells were mixed with 5 × 10^5^ mitomycin C-treated spleen cells plus peptide for 48 h before pulsing with [^3^H]-thymidine and scintillation counting. Shown is data from 1 of 5 assays. “Fold stimulation” (*y*-axis) is the average thymidine incorporation at a given peptide concentration divided by average incorporation into wells pulsed with Flu NP_147–155_. (b) IFN-*γ* expression of LN cells. Expression of IFN-*γ* was assayed as described in “Section 2”. In brief, PLN were isolated from NOD mice (3 weeks, striped bars; 13 weeks, white bars; and diabetic mice, black bars), pulsed with peptides for 48 h, and supernatants were analyzed by ELISA for IFN*γ* production. Shown is data from 1 of 2 assays. (c) RMA-S stabilization assay. Plotted are the ratios of MFI values of peptide-pulsed RMA-S cells to nonpulsed cells stained with either anti-K^d^ or D^b^. For each peptide as indicated the stabilization assay was performed >3 times. (d) Tetramer analysis of NOD spleen cells. Tetramers were used together with anti-CD8 to stain spleen cells of different aged NOD mice (as indicated) as described in “Section 2.” Plotted are tetramer positive cells as a percentage of CD8 cells. (e) Example of tetramer analysis of NOD spleen cells. As example of the data used to generate (d), Anti-Flu NP_147–155 _(top panels), IGRP_206–214_, D*β*H_233–241_, and ZnT8_282–290_ (bottom panels) tetramers were used together with anti-CD8 to stain spleen cells of 13-week-old NOD mice as described in “Section 2.” D*β*H tetramer positive cells were 0.42% of CD8^+^ cells, IGRP tetramer positive cells were 0.66% of CD8^+^ cells, and ZnT8 positive cells were 0.69% of CD8^+^ cells.

*Selection of Candidate CD8* ^*+*^ *T Cell Epitopes*. The literature was reviewed for proteins that induce humoral immunity in human type 1 diabetes; six proteins were selected, five for which candidate epitopes have been previously identified and one, D*β*H, which has been reported as being present in islets [32–34] but has not previously been implicated in type 1 diabetes. Candidates were analyzed by predictive algorithms for MHC Class I-restricted epitopes (9 amino acids, using IEDB, SYFPEITHI, and RANKPEP), and predicted epitopes were synthesized (Table 1) and tested for the ability to stimulate* in vitro* proliferation of total splenocytes obtained from mice of different ages, 3 or 13 weeks, or overtly diabetic (having blood glucose > 250 mg/dL). Most candidate epitopes failed to demonstrate splenocyte proliferation and the level of stimulation was in general modest, possibly reflecting low abundance of antigen-specific cells in spleen (data not shown). Peptides were then tested for stimulation of pancreatic LN cells (Figure 1(a)). Surprisingly the majority of candidate epitopes did not reproducibly stimulate LN proliferation, even peptides that were scored highly by predictive algorithms. However, several previously described epitopes were highly stimulatory: proinsulin_39–47_, IGRP_206–214_, and two epitopes from GAD65. In addition, a candidate peptide of ICAp69, two ZnT8 peptides, and a peptide from D*β*H each stimulated robust LN cell proliferation, predominately in older prediabetic and overtly diabetic mice.

*Assay for Stimulation of IFNγ Production In Vitro.* We next tested candidate peptides for the ability to stimulate secretion of IFN*γ* from PLN (Figure 1(b)). Several peptides stimulated IFN*γ* secretion, usually from cells of mice of the same age that scored in the proliferation assay except for insulin_39–47_ where LN cells from very young mice also responded. Interestingly, in spite of the presumed greater sensitivity of the cytokine production assay, several peptides that stimulated robust LN cell proliferation failed to induce IFN*γ* expression: GAD65_178–186_, ICAp69_78–86_, and ZnT8_158–166_. This could be due to several factors including cell intrinsic lack of IFN*γ* expression by those clones, but no peptide stimulated IFN*γ* secretion that did not also stimulate LN cell proliferation.

*MHC Class I Stabilization Assay*. In order to assess the ability of candidate peptides to bind to MHC Class I, RMA-S stabilization assays were performed [29] and these data are summarized in Figure 1(c). The MHC stabilization data was in general concordant with the IFN*γ* production assay in that, with the exception of ZnT8_158–166_ and to a lesser extent ICAp69_78–86_, candidate epitopes that stimulated IFN*γ* production also strongly stabilized MHC Class I. Collectively considered the three types of assays indicate the existence of CD8^+^ T cells that recognize the predicted cognate epitopes.

*Tetramer Reactivity*. MHC Class I tetramers of the appropriate allele were prepared and NOD splenocytes were assessed by flow cytometry after costaining with anti-CD8 (summarized in Figure 1(d)). These analyses were in general concordant with the IFN*γ* assay in that tetramer-reactive T cells were detected in mice of certain ages coincident with identification of functional* in vitro* T cell activity using cognate peptides.

**Figure 2:**
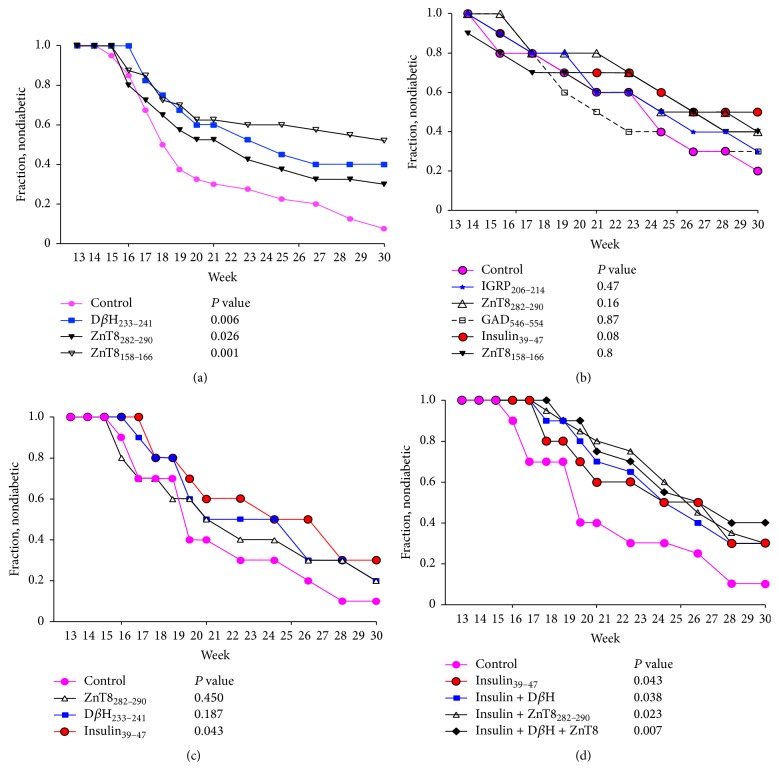
(a) High zone tolerance. Peptides were injected i.p. (*n* = 40 mice per group) beginning at 4 weeks of age as described in “Section 2.” Blood glucose was monitored from age of 13 to 31 weeks. Statistical comparison of peptide-treated groups to the control group receiving PBS at the last week was made by log-rank test (Mantel-Cox). These data are from a single experiment. (b) Peptide plus Zymosan A. Mice (*n* = 10 mice per group) having blood glucose ~170 mg/dL received a single injection of the indicated peptide admixed with Zymosan A as described in “Section 2” and were monitored for blood glucose. Control mice received Flu NP_147–155_ peptide plus Zymosan. The experiment was repeated once and data were combined for statistical comparison to mice receiving control peptide (log-rank test). (c) and (d) DNA plasmid vaccination. Purified plasmids were injected in the quadriceps of NOD mice (*n* = 10 mice per group) beginning at 4 weeks of age as described in “Section 2.” Control mice received purified empty vector. Blood glucose was monitored. (c) shows mice receiving single plasmids and (d) shows mice receiving mixtures of plasmids as indicated. Data for the “control” and “insulin” groups is reproduced in each panel. The experiment was repeated once and data were combined for statistical comparison to mice receiving control plasmid (log-rank test).

*Induction of Epitope-Specific Tolerance*. Using 3 different protocols, we asked if the predicted novel T cell epitopes could be used to influence development of elevated blood glucose. In the first approach, purified ZnT8 and D*β*H peptides were injected at 2-3-week intervals beginning at 4 weeks of age under noninflammatory conditions [23] (Figure 2(a)). In control mice receiving buffer injection, diabetes developed with kinetics and penetrance typical in our mouse facility: ~85% of females had elevated blood glucose by ~30 weeks of age. However disease development was significantly prevented by treatment with ZnT8_282–290_, ZnT8_158–166_, or D*β*H_233–241_ implying that these epitopes may be tolerogenic.

To confirm the tolerizing potential of these epitopes, in the second approach, ZnT8 candidate epitopes were mixed with a stimulator of TLR2 (Zymosan) that has been shown to induce immunological tolerance [31]. In this experiment (Figure 2(b)) the stringency of the tolerizing protocol was increased and we began treatment on older mice that developed elevated blood glucose (~170 mg/dL). In addition, epitopes from previously described antigens IGRP, GAD65, and insulin were separately tested (and control mice were injected with Zymosan plus an irrelevant K^d^ binding peptide, Flu NP_147–155_). Control mice receiving Zymosan plus Flu NP_147–155_ peptide developed disease with slightly slower kinetics than mice receiving only buffer but ultimately the same percentage became diabetic. Although fewer mice treated with ZnT8_282–290_ or ZnT8_158–166_ developed disease compared to controls, the results were not significant. The same was noted for mice receiving IGRP_206–214_, GAD65_546–554_, or insulin_39–47_ peptides leading to the conclusion that coadministration of TLR2 agonists together with these candidate peptide epitopes did not enhance tolerance induction.

**Figure 3:**
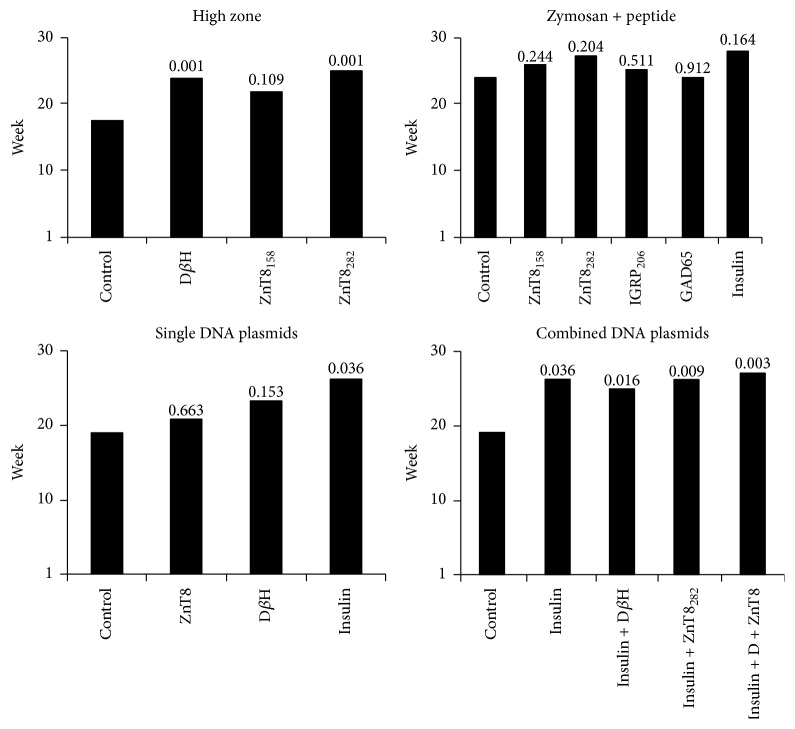
Median age to develop elevated blood glucose after various tolerizing therapies. Data shown in Figures 2(c) and 2(d) were analyzed using the Gehan-Breslow-Wilcoxon test for median age (week) to develop disease. All *P* values are compared within an experiment to the control group and are shown above each bar. The number of mice in each experiment is described in the legend of Figure 2.

The third tolerizing protocol used injection of DNA plasmids encoding antigens containing candidate epitopes [11, 12, 35]. Normoglycemic mice were injected with plasmids encoding either D*β*H or ZnT8, proinsulin, the “empty” plasmid (as negative control), or combinations of these plasmids (Figures 2(c) and 2(d)). As was observed for mice receiving admixture of Zymosan and peptides, injection of individual ZnT8 or D*β*H plasmids reduced disease incidence but not to statistical significance. However, diabetes prevention in mice receiving the proinsulin plasmid was statistically significant (Figure 2(c)). Mice receiving combinations of plasmids were protected to a greater extent than any single plasmid, including proinsulin alone (Figure 2(d)), although comparison of combinations of plasmids to insulin plasmid alone was not statistically significant. Combination of targets was also better at impacting disease compared to single plasmid treatment seen when the data was analyzed for median age of disease development (Figure 3) where each dual combination of plasmid was superior to that of insulin alone, and targeting both ZnT8 and D*β*H in combination with insulin was the most successful.

**Figure 4:**
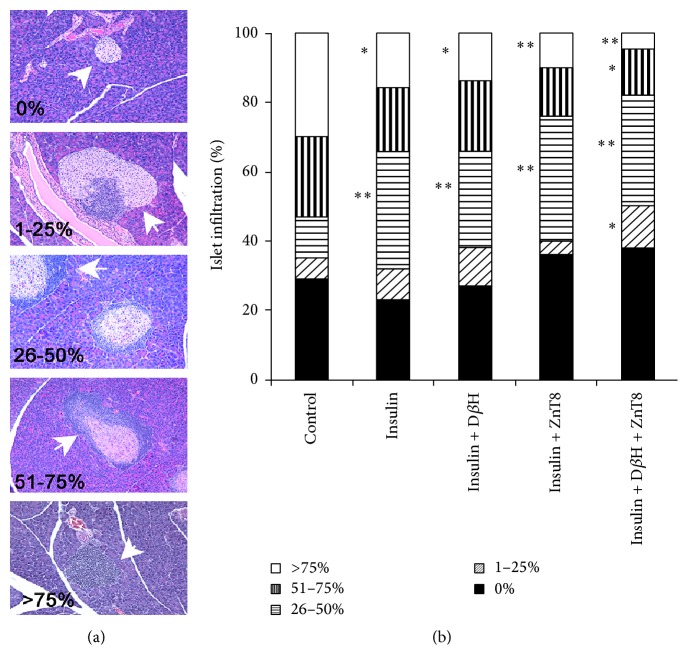
Pancreas histology. Mice from Figure 2(d) were examined after H + E staining of pancreases. Insulitis was assessed by measuring the volume of islet mass obscured by lymphocytic infiltration. Examples of classification of infiltration are shown in (a) and the percentage of islets with 0, 1–25, 26–50, 51–75, and >75% infiltration is shown in (b). 10–20 microscopic fields (containing at least 100 islets) were examined for each treatment group. Groups were compared to control by ANOVA with post hoc comparison. ^*∗*^ *P* = 0.05, ^*∗∗*^ *P* = 0.01.

*Islet Histology of Mice*. In order to characterize the effect of DNA vaccination on islet inflammation, pancreases in mice receiving DNA plasmids that did not show elevated blood glucose at the completion of the experiment were analyzed for extent of lymphocytic infiltration (Figure 4). Reflecting the tolerization data (Figure 2), combinations of plasmids dramatically and significantly reduced the number of islets having severe infiltration (defined as >50%). Interestingly, the number of islets having no insulitis did not decrease, even compared to the control group, and the number of islets having demonstrable insulitis (26–50% of islet area infiltrated) increased in all groups of treated mice. The results from each major experiment are summarized in Table 2.

## 4. Discussion

Immunotherapeutic reversal or downregulation of the diabetogenic adaptive autoimmune response has been pursued as a way to prevent or reverse type 1 diabetes [36]. Since there are more than two dozen non-MHC-linked genes associated with type 1 diabetes, each of which may contribute to disease development and/or maintenance [37], monotherapy that targets any given gene seems unlikely to succeed. Adding more complexity to this task is the considerable antigenic variety revealed by the large number of *β* cell proteins that elicit an immune response many of which are candidates for antigen-specific tolerance induction [3, 38].

Nevertheless, although most experimental immunotherapy trials have been unsuccessful at inducing long-term restoration of regulated insulin secretion, several showed at least partial effects in that the loss of C-peptide is reduced implying enhanced *β* cell function and a reduced rate of disease progression [11, 12, 39]. In the NOD model where experimental design can be more carefully controlled, several approaches to tolerance induction have showed that tolerance to GAD65 and IGRP peptide epitopes can be induced in prediabetic mice, but which has little or minimal effect on disease development [18, 23]. Those observations illustrate that although GAD65 and IGRP are potent autoantigens and immune response to those proteins may significantly contribute to disease maintenance, they do not provide dominant epitopes in the initiation of disease.

Preclinical tolerizing therapy protocols in the NOD model have showed that insulin is a primary autoantigen and initiates type 1 diabetes. This was convincingly demonstrated when a key residue in the insulin B_9–23_ epitope was replaced; disease is prevented [20]. Even though anti-insulin T cells are produced very early in disease, T cells having other, noninsulin specificities accumulate in islets as NOD mice age. This occurs before overt disease develops [40] leading to the notion that pathogenic T cells having specificities for other antigens contribute in a substantial way to disease. This probably underlies the observation in an experimental trial that, even with successful reduction of insulin-specific T cells following tolerization, insulin independence was not achievable [12] (in addition, results from one phase III trial that targeted a HSP peptide (Diapep277) that was supported by encouraging early stage data was subsequently retracted [41]). Considered collectively these data suggest that an unknown number of *β* cell-reactive T cells likely need to be simultaneously rendered inactive in order to reduce or eliminate islet inflammation and destruction of function. Based upon data achieved by screening candidate peptides with patient peripheral blood lymphocytes that identified dozens of peptides having stimulatory activity, this appears likely. For example, for the antigen ZnT8 Enee and colleagues have identified nine candidate epitopes [42] although the existence of a given T cell with islet reactivity, even prior to development of type 1 diabetes, does not establish if that T cell is causally associated with disease, evidence which needs to be achieved before consideration as part of an immunotherapeutic protocol.

Conclusive proof for a role of any given peptide in development of diabetes requires being able to influence the incidence or progression of disease by gene deletion or epitope-specific induction of tolerance. Our results that identify new *β* cell diabetogenic epitopes illustrate this point and argue in favor of continued effort at antigen identification. Our approach to identification of diabetogenic epitopes focuses on candidate peptides that have high HLA binding affinity (which are then screened for biological activity) which appears to be valid in the sense that several novel diabetogenic epitopes have been identified. However, it remains possible that low affinity candidate epitopes that were not strong binding peptides may nonetheless be associated with disease, but corresponding synthetic peptides were not tested for tolerizing potential* in vivo* because of exclusion in preliminary experiments. Screening all candidate peptides using* in vivo* tolerizing experiments, and in multiple combinations (which appears a likely requirement for successful therapy of patients), is a definitive, but challenging and unfortunately perhaps untenable, approach for epitope identification. Those candidate epitopes that may be excluded from further consideration on the basis of low responses in peptide-based* in vitro* assays are contained within the cDNA plasmids used for combinatorial* in vivo* tolerizing experiments and thus may contribute to the biological effects of plasmid-based therapy. A major future goal for this experimental approach is to determine if plasmid vaccination using any given diabetogenic antigen results in development of multiple T cells having different specificities and determination if those T cells contribute to tolerance induction.

The mechanism of tolerance induced by the ZnT8_282–290_ and D*β*H_233–241_ epitopes is currently unknown but, since tetramer-reactive cells were detected in tolerant normoglycemic mice at >30 weeks of age (dns), likely does not involve clonal deletion as has been suggested for plasmid-based targeting of proinsulin-reactive T cells in a recent human trial [12]. In addition, total numbers of PLN Treg are not changed in treated mice (dns). Possibilities on how tolerance is developed include activation of antigen-specific Treg that suppress the diabetogenic phenotype, functional anergy, development of cryptic (subdominant) low affinity T cells that block the effector phase of diabetogenic clones, or deviation of cytokine expression in the cognate T cell. All these mechanisms for tolerance induction are testable in future studies.

## Figures and Tables

**Table 1:** The amino acid numbers of the primary translation product of each peptide are indicated. Asterisk for insulin_39–47_ indicates amino acid substitution (G>V) at position 9 that was shown to enhance MHC binding stability [22].

Insulin	D^b^	Insulin	K^d^
2–10	ALLVHFLPL	6–14	HFLPLLALL
3–11	LLVHFLPLL	22–30	TQAFVKQHL
22–30	TQAFVKQHL	39–47	LYLVCGERV^*∗*^
31–39	CGPHLVEAL	71–79	SPGDLQTLA
70–78	GSPGDLQTL	92–100	QCCTSICSL
95–103	TSICSLYQL		

IGRP	D^b^	IGRP	K^d^

33–41	GDPRNIFSI	18–26	DYRTYYGFL
207–215	YLKTNVFLF	41–49	IYFPLWFQL
216–225	FALGFYLLL	123–131	WYVMVTAAL
251–259	KWCANPDWI	151–159	SFLWSVFWL
258–265	GLVRNLGVL	206–214	VYLKTNVFL
271–279	FAINSEMFL	255–263	PFAGLVRNL
296–304	CALTSLTTM	392–401	RLLCALTSL
311–319	KIPTHAEPL		

ICAp69	D^b^	ICAp69	K^d^

62–70	FHSIQRTCL	14–22	RFAQDKSVV
199–207	LAKKNFDKL	78–86	LYQKRICFL
221–229	SRCNLLSHM	111–119	MMQATGKAL
258–266	YQPYEFTTL	189–197	KFRKVQTQV
390–398	AAVFGDDQL	285–293	SWRENREAV
425–433	LLDQNMKDL		

GAD65	D^b^	GAD65	K^d^

84–92	KGDVNYAFL	178–186	YFNQLSTGL
151–160	DQPQNLEEI	206–214	TYEIAPVFV
243–251	GAISNMYAM	340–348	VYGAFDPLL
389–397	SVTWNPHKM	436–444	SYDTGDKAL
551–559	GDKVNFFRM	546–554	SYQPLGDKV

ZnT8	D^b^	ZnT8	K^d^

28–36	QKPVNKDQC	7–15	TYLVNDQAT
76–84	ASAICFIFM	112–120	SFLLSLFSL
77–185	SAICFIFMV	158–166	LYLACERLL
174–182	CAASAICFI	237–245	IYFKPDYKI
186–194	AANIVLTMI	282–290	SYNSVKEII
306–314	SLTVNQVIL	325–333	DSQSVRTGI

D*β*H	D^b^	D*β*H	K^d^

64–72	ELSWNVSYV	233–241	TYWCYITEL
97–105	GEMENADLI	363–371	RYDAGIMEL
411–419	FASQLHTHL	420–428	TGRKVVTVL
548–556	WNSFNRNML	471–479	TYNTENKTL
578–586	PGEWNLQPL	498–506	YYPQTELEL

**Table:** (a)

Antigen	aa	Candidate peptides that stimulate			Candidate peptides that stimulate		
		pancreatic LN cell proliferation^a^			pancreatic LN cell secretion of IFN*γ* ^b^		
		3 weeks	13 weeks	Diabetic	3 weeks	13 weeks	Diabetic
Control	147–155	−	−	−	−	−	−
Insulin	39–47	−	+	+	+	+	+
Gad65	178–186	−	+	−	−	−	−
Gad65	546–554	−	+	+	−	+	+
IGRP	206–214	−	+	+	−	+	+
ICAp69	78–86	−	−	+	−	−	−
ZnT8	158–166	−	+	+	−	−	−
ZnT8	282–290	−	+	+	−	+	+
D*β*H	233–241	−	+	+	−	+	+

**Table:** (b)

Antigen	RMAS^c^	Tetramer binding^d^			Tolerance induction^e^		
		3 weeks	13 weeks	Diabetic	Purified peptide	Peptide + Zymosan A	Plasmid
Control	+	−	−	0.04			
Insulin	+	0.03	0.72	0.89	nt	+	+
Gad65	−	0.02	0.1	0.1	nt	nt	nt
Gad65	+	0.03	0.39	0.14	nt	ns	nt
IGRP	+	−	0.63	0.57	nt	ns	nt
ICAp69	+	−	−	−	nt	ns	nt
ZnT8_158_	+	−	0.20	0.26	+	ns	nt
ZnT8_282_	+	−	0.69	0.60	+	ns	ns
D*β*H	+	0.09	0.41	0.32	+	nt	ns
Ins + D*β*H						nt	+
Ins + ZnT8						nt	+
Ins + D*β*H + ZnT8						nt	+

Control peptide is influenza NP.

“−” and “+” refer to whether peptide surpasses cutoff assignment.

“nt”: not tested.

“ns”: not significant.

a: scoring cutoff is >5-fold over control.

b: scoring cutoff is >500 pg/mL. (control is <100 pg/mL).

c: scoring cutoff is >2-fold difference in MFI.

d: % of pancreatic LN CD8^+^ T cells.

e: “+” indicates scoring cutoff has statistically significant *P* value versus control peptide.
